# DataDTA: a multi-feature and dual-interaction aggregation framework for drug–target binding affinity prediction

**DOI:** 10.1093/bioinformatics/btad560

**Published:** 2023-09-09

**Authors:** Yan Zhu, Lingling Zhao, Naifeng Wen, Junjie Wang, Chunyu Wang

**Affiliations:** Faculty of Computing, Harbin Institute of Technology, Harbin 150001, China; Faculty of Computing, Harbin Institute of Technology, Harbin 150001, China; School of Mechanical and Electrical Engineering, Dalian Minzu University, Dalian 116600, China; Department of Medical Informatics, School of Biomedical Engineering and Informatics, Nanjing Medical University, Nanjing 211166, China; Faculty of Computing, Harbin Institute of Technology, Harbin 150001, China

## Abstract

**Motivation:**

Accurate prediction of drug–target binding affinity (DTA) is crucial for drug discovery. The increase in the publication of large-scale DTA datasets enables the development of various computational methods for DTA prediction. Numerous deep learning-based methods have been proposed to predict affinities, some of which only utilize original sequence information or complex structures, but the effective combination of various information and protein-binding pockets have not been fully mined. Therefore, a new method that integrates available key information is urgently needed to predict DTA and accelerate the drug discovery process.

**Results:**

In this study, we propose a novel deep learning-based predictor termed DataDTA to estimate the affinities of drug–target pairs. DataDTA utilizes descriptors of predicted pockets and sequences of proteins, as well as low-dimensional molecular features and SMILES strings of compounds as inputs. Specifically, the pockets were predicted from the three-dimensional structure of proteins and their descriptors were extracted as the partial input features for DTA prediction. The molecular representation of compounds based on algebraic graph features was collected to supplement the input information of targets. Furthermore, to ensure effective learning of multiscale interaction features, a dual-interaction aggregation neural network strategy was developed. DataDTA was compared with state-of-the-art methods on different datasets, and the results showed that DataDTA is a reliable prediction tool for affinities estimation. Specifically, the concordance index (CI) of DataDTA is 0.806 and the Pearson correlation coefficient (R) value is 0.814 on the test dataset, which is higher than other methods.

**Availability and implementation:**

The codes and datasets of DataDTA are available at https://github.com/YanZhu06/DataDTA.

## 1 Introduction 

Drug discovery offers significant potential benefits to patients and the pharmaceutical industry. Due to the rapid development of proteomics and the increasing number of target molecules for drug action, computational methods have penetrated all aspects of drug research and development as an alternative to wet-laboratory experimental determination. In particular, in silico methods for drug–target binding affinity (DTA) prediction have attracted great attention and have developed rapidly.

Binding affinity provides information on the interaction strength between a drug–target pair, usually expressed as the dissociation constant (*K*d), inhibition constant (*K*i), or half-maximal inhibitory concentration (*IC*50) (Cer *et al.* 2009). Virtual screening through physics-based methods, such as molecular docking and pharmacophore modeling (Lavecchia 2015, Vuorinen and Schuster 2015), has been popularly used in binding affinity prediction of small molecules interacting with proteins. Although virtual screening is faster and more effective compared than wet-laboratory experimental technology, some barriers, such as the high-calculation cost and operational complexity have prevented its practical use (Kairys *et al.* 2019). Alternatively, data-driven approaches, such as quantitative feature relationship methods with machine learning (ML) and deep learning (DL) technologies, have been widely studied over the past few decades. DL is outstanding not only in terms of computer vision (Wang *et al.* 2021a) and natural language processing (Otter *et al.* 2021), but also when applied to bioinformatics (Cao *et al.* 2020), computational biology (Tang *et al.* 2019) and biomedicine (Cao *et al.* 2018). It can extract important characteristics automatically and has successfully dealt with a variety of problems in the drug discovery process, such as de novo molecular design (Abbasi *et al.* 2020), protein-binding pocket identification (Shi *et al.* 2020, Zhang *et al.* 2020) and biomolecular interaction analysis (Pan *et al.* 2019, Song *et al.* 2022).

Some progress has also been made in applying DL methods to DTA prediction tasks. Depending on which protein–compound complex structures are used, DL methods applied to DTA prediction can be classified as native complex structure-based methods or complex structure-free methods. Native complex structures are those in which protein–ligand 3D complex structures are verified by experiments. For example, Stepniewska-Dziubinska *et al.* (2018) proposed the DL model Pafnucy, which represents the input structure as a 3D grid, utilizes a 3D convolution to produce a feature map of the representation, and finally uses dense layers to predict affinity values. Cang and Wei (2017) developed TopologyNet, which employs the element-specific persistent homology method (representing 3D complex geometry via 1D topological invariants) with deep convolutional neural networks (CNNs). Wang *et al.* (2021) developed DeepDTAF, which integrates protein sequence information, protein-binding pockets, and the simplified molecular input line entry system (SMILES) as well as the secondary structural properties of proteins and pockets, and feeds these input features into embedding layers and dilated or traditional convolution layers. OnionNet-2 (Wang *et al.* 2021) is a 2D-CNN based regression model to predict protein–ligand binding affinities, which used the protein–ligand complexes for training and adopts the rotation-free residue-atom-specific contacts in multiple distance shells to describe the protein–ligand interactions. Wang *et al.* (2022) proposed PointTransformer, which applied the point cloud-based neural network structures PointNet and PointTransformer for the prediction of protein–ligand affinity trained on the protein–ligand complexes dataset. Obviously, the requirements of native complex structure information approaches limit the scope of their application. In contrast, complex structure-free methods do not require prior knowledge of complex structures and thus have emerged in large numbers. For instance, Öztürk *et al.* (2018) proposed the DL-based method DeepDTA with CNN to learn representations from protein sequences and ligand SMILES, and their WideDTA (Öztürk *et al.* 2019) used four different textual pieces of information to encode the drug SMILES and protein sequences, and processed information using CNN blocks. Abbasi *et al.* (2020) developed a deep learning-based method, DeepCDA, which combines CNN and long short-term memory (LSTM) layers into a unified framework to encode the local and global temporal patterns and which uses a two-sided attention mechanism to fuse the compound and protein descriptors. Nguyen *et al.* (2021) proposed a method called GraphDTA, in which drugs are represented as graphs with a feature map and an adjacent matrix and are processed by graph neural networks, including graph convolutional networks (GCNs), graph attention networks (GATs), and graph isomorphic networks (GINs), while proteins are encoded by CNN blocks to predict DTAs. MATT_DTI was designed by Zeng *et al.* (2021), using a relation-aware self-attention block and a CNN block in the drug representation learning process and a CNN block in the protein representation learning processes. FusionDTA was built by Yuan *et al.* (2022), which applied a pretrained transformer to generate the distributed input representation, and used a multihead linear attention mechanism to aggregate global information based on the attention score and to replace the pooling operation. Ru *et al.* (2022) treated DTA prediction as a search ranking task and proposed NerLTR-DTA, which applied the neighbor relationship of similarity and sharing to extract features, and used a ranking framework with regression attributes to predict affinity values and priority order of query drug/target and its related proteins/compounds. Recently, Hua *et al.* (2023) developed MFR-DTA with a BioMLP block to extract individual features from sequence elements and an Elem-feature block to refine extracted features, and a Mix-Decoder block to extract the drug–target interaction features for binding regions and affinities prediction.

Despite significant research efforts and some success, the DTA prediction problem still remains unsolved because existing complex structure-free methods fail to collect and leverage key DTA-related information effectively. For instance, it is widely known that protein-binding pockets are crucial regions for protein–ligand interactions and are commonly used as targets for disease treatment (Le Guilloux *et al.* 2009). Drug–target interactions are frequently driven by key hotspot residues in the concave regions of biomolecular surfaces (Tubiana *et al.* 2022). Therefore, binding pocket information can serve as an enhancement of protein data to assist affinity prediction. However, because binding pocket prediction is regarded as another prediction task, pocket information is commonly ignored in the current DTA prediction framework to our knowledge. Fortunately, many pocket detection tools have been modeled because pocket prediction is crucial for large-scale protein function prediction and protein druggability (Le Guilloux *et al.* 2009, Volkamer *et al.* 2010, Zhu and Pisabarro 2011, Abibi *et al.* 2014, Rooklin *et al.* 2015). Predicting protein druggability based on the 3D structure of protein targets is mainly divided into two steps: identification of binding pockets for drug-like molecules and evaluation of the druggability of the pockets. Evaluation of the structure of many analyzed proteins and drug-like molecular complexes has revealed that the binding sites generally feature large and flat binding surfaces with good hydrophobicity (Katigbak *et al.* 2020). Additionally, experimental studies, have shown that large conformational changes in the binding region, such as the transition between open and closed states of the binding pocket, and the local secondary structure changes induced by the ligand, are associated with the ligand binding affinity (Yasuda *et al.* 2022). Based on geometric characteristics and the advancement of computer technology, pocket recognition work gradually overcome the limitations of a series of traditional methods. In addition, the development of computational methods for predicting 3D protein structures from the protein sequences has become increasingly mature, with improved speed, accuracy and efficiency. For example, AlphaFold2 (Jumper *et al.* 2021) is a combination of the bioinformatics and physical approaches, which can regularly predict protein structures with atomic accuracy even in cases in which no similar structure is known. There has been considerable progress in developing protein-binding pockets detected by computational predictive tools from the 3D structure of proteins instead of native complexes derived from traditional experimental methods.

Currently, many researchers prefer using well-pretrained encoder models with large amounts of unlabeled data and subtle training tasks to further generate latent-space vectors as molecular representations (Ye *et al.* 2021, Zeng *et al.* 2022). Using well-pretrained encoder models with large amounts of unlabeled data and subtle training tasks is indeed beneficial for generating molecular representations. These models have more flexibility and transferability in generating latent-space vectors as molecular representations, which can improve downstream task performance (Jin *et al.* 2018). Moreover, pre-trained encoder models automatically learn the most important features from data, making them well-suited to support various inference tasks for molecular properties. As a result, high-level features of molecular structures that may be challenging to identify through manual feature engineering can be captured (Goh *et al.* 2017). However, latent-space representations overlooked many stereochemical information such as dihedral angles and chirality (Blondel and Karplus 1996, Nguyen and Wei 2019), and the representations lack specific physical and chemical knowledge, making it difficult to accurately describe attributes related to specific tasks. For instance, in many drug-related properties, van der Waals interactions may play a more significant role than covalent interactions, thus requiring their consideration when describing these properties (Chi *et al.* 2010). Designing molecular descriptors can provide more useful information in this regard. Among them, algebraic graph-based fingerprints (AG-FPs) are an important method that utilizes the element-specific weighted colored algebraic graphs to generate intrinsically low-dimensional molecular representations while preserving essential physical/chemical information and physical insight (Chen *et al.* 2021). This approach shows great promise for better application in the description and prediction of molecular structures.

In this work, we developed a novel neural network framework, DataDTA (Deep information aggregation for DTA prediction), to predict drug–target binding affinities by combining the advantages of various digital representations and a fusion mechanism. These representations are intrinsically generated from low-dimensional molecular data and SMILES strings of compounds, as well as binding pocket descriptors and raw sequences of proteins. To effectively capture the feature vectors not only at the bit-wise level but also at the vector-wise level simultaneously, the fusion strategy with a highway block and a multihead attention block was used to model the complex interaction. The proposed DataDTA was applied to three test datasets from the PDBbind database involving a collection with experimentally measured binding affinity data for biomolecules complexed from the Protein Data Bank (PDB). Extensive validation and comparison results suggest that DataDTA is a powerful and effective computational method for predicting DTA.

In summary, the main contributions of DataDTA include:

- Protein-binding pocket descriptors for effective protein feature mining. The aim is to provide valuable information about key binding sites.
- The AG-FPs as compound structure descriptors for valuable compound presentation. It can effectively capture topological and physical/chemical information about the molecules.
- A fusion strategy with a highway block and a multihead attention block for multiscale interaction information. It can integrate and capture interaction information of drugs and targets at the bit-wise level and at the vector-wise level simultaneously.

## 2 Materials and methods

### 2.1 Overall framework

**Figure 1.**
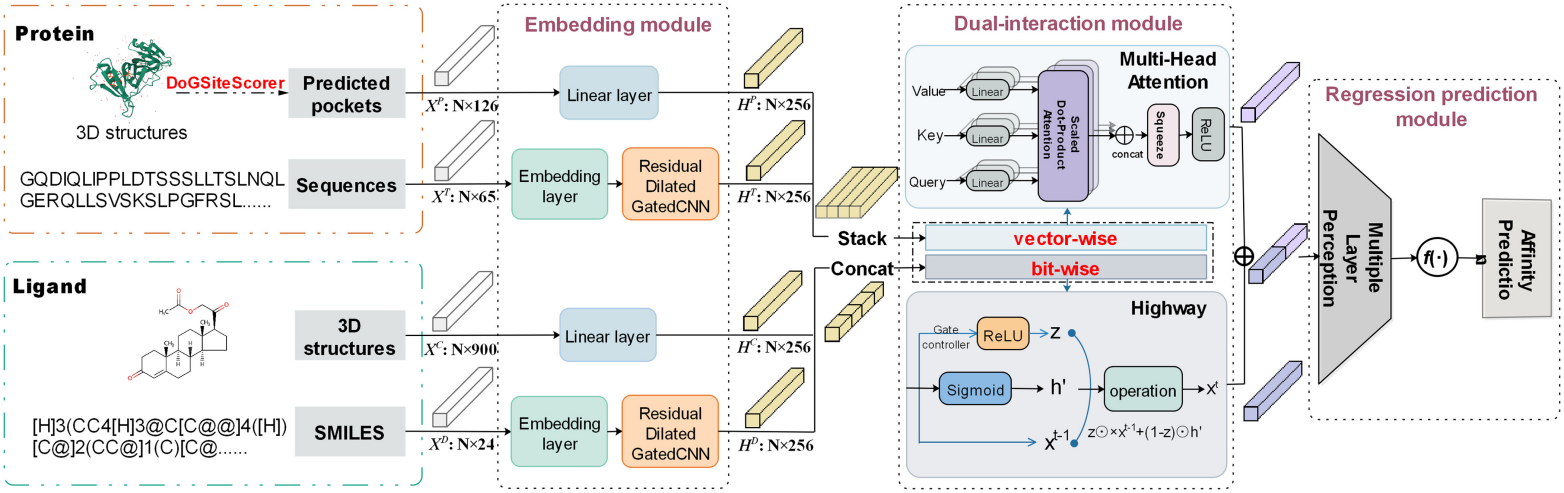
Overview of DataDTA.

We proposed a computational framework named DataDTA to predict binding affinities between drugs and targets. There were four major steps involved in the development of DataDTA, including data preparation, input encoding, neural network construction, and model evaluation. In the first step, we collected benchmark datasets that contained structure files of these sequence data for model training and performance validation. In the second step, sequence and structure information of drugs and targets were converted into digital matrices to prepare the input data for the neural network. In the third step, we build a multiview deep learning framework, which is mainly composed of a CNN module, a dual-interaction aggregation module that includes a highway block and a multihead attention block, and a linear transformation output module. In the fourth step, we assessed and evaluated the predictive performance of DataDTA based on test datasets. In summary, we developed a method named DataDTA, which uses four input representations and a dual-interaction aggregation neural network to carry out DTA prediction tasks, as shown in Fig. 1.

### 2.2 Datasets

We trained and evaluated our proposed model on the PDBbind dataset following the previous methods (Stepniewska-Dziubinska *et al.* 2018, Wang *et al.* 2021). Three datasets (the general set, the refined set, and the core 2016 set) from PDBbind version 2016 and two additional test datasets (test105 and test71) from the PDB were used in this work. The statistical summary of the datasets is provided in Table 1. The quantities of original protein–target pairs in the general set, refined set, and core 2016 set were 9226, 4057, and 290, respectively. To guarantee that the data did not overlap and to facilitate model comparison, the datasets were processed in the same way as the previous work (Stepniewska-Dziubinska *et al.* 2018, Wang *et al.* 2021). Thus, the final quantities of protein–target pairs were 9221, 3685, and 290 in the general set, refined set and core 2016 set, respectively. Then, 1000 protein–target pairs were randomly selected in the refined set to serve as the validation set. The remaining complexes in the refined set and the whole general set were together used as the training set. The entire core 2016 set was used as the test dataset. Overall, there were 11 906 training samples, 1000 validation samples, and 290 test samples. The three datasets, as well as two additional test datasets containing 71 and 105 test samples, were used in our work.

**Table 1.** Benchmark results of the cascade oscillators model.

Dataset	Source	Original protein–target pair quantity	Final protein–target pair quantity	Training samples	Validation samples	Test samples
General set	PDBbind	9226	9221	9221	–	–
Refined set	PDBbind	4057	3685	2685	1000	–
Core 2016 set	PDBbind	290	290	–	–	290
Test105 set	PDB	105	105	–	–	105
Test71 set	PDB	71	71	–	–	71

We set the fixed lengths to 120 for SMILES strings and to 1000 for protein sequences in these datasets. Longer sequences were truncated and shorter sequences were padded with zeros to the fixed lengths.

### 2.3 Input representation

In this study, we used four different information sources to model proteins and ligands. For sequences, integer/label encoding that uses integers for the categories to represent drug and protein raw inputs was applied since the previous published works have shown that integer/label encoding is an effective approach (Wang *et al.* 2021, Yuan *et al.* 2022). The label encoding for drug SMILES strings is given as $X^{D}=\left\{x_{1}^{D},x_{2}^{D},x_{3}^{D},\ldots ,x_{120}^{D}\right\}\in R^{V_{D}}$. Similarly, the label encoding for protein sequences is represented as $X^{S}=\left\{x_{1}^{S},x_{2}^{S},x_{3}^{S},\ldots ,x_{1000}^{S}\right\}\in R^{V_{P}}$, where $V_{D}$ and $V_{S}$ are the vocabulary sizes in the format of SMILES and amino acids, respectively.

Besides, we explored additional information about drugs and proteins. For a given drug molecular structure, AG-FPs are low-dimensional molecular representations derived from the element-specific weighted colored algebraic subgraphs, which can essentially capture topological and physical/chemical information about the molecules (Chen *et al.* 2021). Here, we used AG-FPs to characterize the properties of a molecule for each drug. Ten chemical elements including “H”, “C”, “N”, “O”, “F”, “P”, “S”, “Cl”, “Br”, “I”, and the element-specific weighted Laplacian matrix were taken in this work (Chen *et al.* 2021). This enabled a set of element-specific weighted colored Laplacian matrix-based molecular descriptors to be directly constructed by the statistics of nontrivial eigenvalues, i.e. summation, minimum, maximum, average, and standard deviation of nontrivial eigenvalues. Finally, a 900D vector was obtained and the AG-FPs representations of drug molecular can be expressed as $X^{C}=\left\{x_{1}^{C},x_{2}^{C},x_{3}^{C},\ldots ,x_{900}^{C}\right\}\in R^{900}$.

Furthermore, protein-binding pockets were introduced as input. First, the webpage of predicting binding sites and estimating their druggability (DoGSiteScorer) (Volkamer *et al.* 2010) in Proteins*Plus* (https://proteins.plus) was applied to predict the pockets for each protein structure file. Then, 42 descriptors were extracted from each pocket consisting of 7 size and shape descriptors (volume, enclosure, surface, depth, ellipsoid main axis ratio *c*/*a*, ellipsoid main axis ratio *b*/*a*, surface/volume), 5 functional group descriptors (hydrogen bond donors, hydrogen bond acceptors, metals, hydrophobic interactions, and hydrophobicity ratio), 6 element descriptors (pocket atoms, carbons, nitrogens, oxygen atoms, sulfurs, and other elements), and 4 amino acid compositions (polar amino acid ratio, polar amino acid ratio, positive amino acid ratio, and negative amino acid ratio) as well as 20 amino acid composition descriptors (alanine, arginine, asparagine, aspartic acid, cysteine, glutamine, glutamic acid, glycine, histidine, isoleucine, leucine, lysine, methionine, phenylalanine, proline, serine, threonine, tryptophan, tyrosine, and valine). Ultimately, we retained the predicted top-3 pocket data (using zero filling processing for less than three pockets), because it is reported that in 93% of the PDBbind dataset DoGSiteScorer (Volkamer *et al.* 2010) ranks the cavity containing at least one ligand atom among the top-3 pockets. Thus, a total of 126 pocket features were obtained to describe the local “hotspot” region information. Accordingly, the pocket descriptors can be denoted as $X^{P}=\left\{x_{1}^{P},x_{2}^{P},x_{3}^{P},\ldots ,x_{126}^{P}\right\}\in R^{F}$, where *F* denotes the feature dimension of each pocket.

### 2.4 Network architecture design of DataDTA

The complete network architecture and hyperparameters used in the model are shown in Fig. 1 and Supplementary Table S1. The first part is the embedding module in the network architecture of DataDTA. The CNN submodule is designed to construct the basic blocks of the encoder layer for the protein and drug sequence representations. We also feed AG-FPs and pocket descriptors into the linear layer. The second part is the dual-interaction module. The four intermediate features obtained above are stacked and input into this module to learn the interaction. The last part is the regression prediction module, which feeds the obtained representation into the linear layers to obtain the predicted binding affinity.

For the parameters, Supplementary Table S1 lists the specific values used by the model in detail, and mean squared error (MSE) was adopted as the loss function (see Section 2.5 for details). To achieve better training and optimal predictive performance, we also intersperse the network with layer normalization and dropout mechanisms (Lin *et al.* 2022). In the following sections, we will describe the major parts of DataDTA in detail.

#### 2.4.1 Embedding module

The central part of the CNN submodule is a stack of residual blocks, which have been widely used in computer vision and bioinformatics because the shortcut connection added in residual blocks makes the training of extremely deep CNNs possible (Kandel *et al.* 2021, Roy *et al.* 2022). In our residual blocks, traditional convolution was replaced by dilated convolution that supports exponential expansion of the receptive field without loss of resolution or coverage, to capture long-range interactions for protein sequences $X^{S}$ and drug SMILES strings $X^{D}$ (Yu and Koltun 2016). Let F: $Z^{2}$ → R be the discrete function, *k*: $\Omega_{1}$→R be the discrete filter, *l* be the dilation rate, and *s* and *t* be subscripts of element vectors. With this notation, the dilated convolution operator ${*}_{i}$ that we follow is defined as:

(1) $$(F{*}_{i}k)(p)=\sum_{s+lt=p}F(s)k(t).$$

Thus, the discrete function can be defined as follows:

(2) $$F_{i+1}=F_{i*2^{i}}\;k_{i}\;\mathrm{for}\;i\;=\;0,\;1,\;\ldots ,\;n-2.$$

The outputs of the CNN module for targets and drugs are defined as $H^{S}=\left\{h_{1}^{S},h_{2}^{S},x_{3}^{S},\ldots ,x_{m}^{S}\right\}\in R^{m}$and $H^{D}=\left\{h_{1}^{D},h_{2}^{D},x_{3}^{D},\ldots ,x_{m}^{D}\right\}\in R^{m}$, respectively, where *m* is the final output dimension of 256.

In addition, the pocket descriptors $X^{P}$ and compound structure representation $X^{C}$ have the same dimensions as the drug and target information processed by the CNN module. The linear layer is used to control the output of pocket and compound structure information. The outputs of pocket and compound can be recorded as $H^{P}=\{h_{1}^{P},h_{2}^{P},h_{3}^{P},\ldots ,h_{m}^{P}\}\in R^{m}$ and $H^{C}=\{h_{1}^{C},h_{2}^{C},h_{3}^{C},\ldots ,h_{m}^{C}\}\in R^{m}$, where *m* is the output dimension 256.

(3) $$H^{P}=\mathrm{Linear}(126,\;256).$$

(4) $$H^{C}=\mathrm{Linear}(900,\;256).$$

#### 2.4.2 Dual-interaction module

We use the highway network and the multihead attention mechanism for comprehensive processing in the late fusion stage to effectively capture the information of proteins and compounds from four different sources. The embedding features were stacked horizontally and vertically for the multihead attention module and highway network module, respectively, to allow the network to learn different dependencies, aiming for them to learn simultaneously at the bit-wise level and vector-wise level. As shown in the following formula, these resources are combined as the input of the two modules, and $x_{1}$ and $x_{2}$ are then put into two modules to obtain the outputs $o_{1}$ and $o_{2}$. The two modules are described in detail in the following sections.

(5) $$x_{1}=\mathrm{Concat}(H^{D},\;H^{S},\;H^{P},\;H^{C}).$$

(6) $$x_{2}=\mathrm{Stake}(H^{D},\;H^{S},\;H^{P},\;H^{C}).$$

(7) $$o_{1}=\mathrm{Highway}(x_{1}).$$

(8) $$o_{2}=\mathrm{MultiHeadAttention}(x_{2}).$$

##### 2.4.2.1 Highway block

The highway network relies on a gating mechanism that learns how to regulate the information flow through the network. It enables information to pass through the layers of the deep neural network at high speed without hindrance, which effectively slows down the gradient problem. By stacking four features obtained earlier vertically, highway block can learn dependencies between different input features. This block implements skip connections at the network level, allowing different bits of the embedding feature to have different contributions in the network. Therefore, the block operates at the bit-wise level, the network learns to adaptively transform the input based on the properties of the data, allowing it to learn rich representations of the input features. Highway operations essentially use element-wise multiplication and addition operations to implement what is commonly known as the forward pass of the operation (Srivastava *et al.* 2015). A highway network is defined as:

(9) $$y_{1}=H\left(x,W_{H}\right)\;T\left(x,\;W_{T}\right)+x\cdot C(x,\;W_{C})$$

where, $W_{H}$, $W_{T}$, and $W_{C}$ represent weight parameters, *H*(·) is a nonlinear activation function, and the rectified linear unit (ReLU) function was used in this work. *T*(·) refers to a transform gate taken as a sigmoid function, and *C*(·) refers to a carry gate taken as 1−*T*(·), these act as gates and regulate the flow of information through nonlinear *H*(·) and skip path *x*.

##### 2.4.2.2 Multihead attention block

The improved version of multihead attention obtained by improving traditional attention is the core component of models, such as Transformer (Vaswani *et al.* 2017) and BERT (Devlin *et al.* 2018). In this block, the embedding features were stacked horizontally to create a single input matrix. This module aims to learn at the vector-wise level. It operates on the vector representation of input features and aggregates information in the vector space by introducing multiple attention heads, which simultaneously learn their weights and are weighted averaged to capture higher-level semantic information of the input features. This approach better handles global relationships between input features rather than just the relationships between individual bits of the input feature. Consequently, multihead attention module learns at the vector-level using the relationships between vectors to perform calculations and inference (Liu *et al.* 2020). Multihead attention can be expressed as:

(10) $$\mathrm{MultiHeadAttention}\left(Q,\;K,\;V\right)=\mathrm{Concat}\left({\mathrm{head}}_{i},\;\ldots ,\;{\mathrm{head}}_{h}\right){\;W}^{0},$$

where

(11) $${\mathrm{head}}_{i}=\mathrm{Attention}\left({QW}_{i}^{Q},KW_{i}^{K},{VW}_{i}^{V}\right).$$

(12) $$\mathrm{Attention}\left(Q,K,V\right)=\mathrm{softmax}\left(\frac{{QK}^{T}}{\sqrt{d_{k}}}\right)V.$$

where *Q*, *K*, and *V* represent *Query*, *Key*, and *Value*, respectively, which are projections obtained by *h* different linear transformations, $W^{0}$ and ${\left\{W_{i}^{Q},\;W_{i}^{K},\;W_{i}^{V}\right\}}_{i=0}^{h}$ are weight matrices, and $d_{k}$ is the normalized coefficient. Finally, the output of the multihead attention block is defined as:

(13) $$y_{2}=H\left(\mathrm{MultiHeadAttention}\left(Q,\;K,\;V\right)\right),$$

where *H*(·) is also a ReLU activation function here.

#### 2.4.3 Regression prediction module

Three linear layers were used in the network to measure prediction data. Specifically, the outputs *o*_1_ and *o*_2_ were accumulated and the final affinity values were estimated through fully connected layers. The formula is as follows:

(14) o=Linear(256 * 4, 1024, 521, 1).

### 2.5 Performance evaluation

In this work, five performance measures were applied to evaluate and compare the predictive performance of DataDTA and existing methods. These measures included the concordance index (CI), mean squared error (MSE), root mean square error (RMSE), Pearson correlation coefficient (R), and standard deviation (SD) in regression (Gonen and Heller 2005, Li *et al.* 2014).

CI is based on probability between the predicted values and the ground truth values for two randomly selected drug–target complexes in a specific order, which can evaluate the degree of fit of the model. The CI value varies between 0 and 1, and the larger the value is, the better the prediction performance of the model. CI is defined as follows:

(15) $$\mathrm{CI}=\;\frac{1}{Z}\sum_{y_{i}>y_{j}}h(p_{i}-p_{j}),$$

where *p_i_* is the prediction value for the larger binding affinity value *y_i_*, and *p_j_* is the prediction value for the smaller affinity value *y_j_*. *Z* is the normalization constant and the step function *h(x)* equals 1, 0.5, and 0 for *x *>* *0, *x *=* *0 and *x *<* *0, respectively.

The MSE value evaluates the prediction accuracy of the model, that is, the difference between the predicted values and the real values. The lower the MSE values are, the better the model. MSE is differentiable, meaning that a derivative exists at every point; hence, it can be used as a loss function. This measure is calculated as follows:

(16) $$\mathrm{MSE}=\;\frac{1}{n}\sum_{i=1}^{n}{\left(p_{i}-y_{j}\right)}^{2},$$

where *p_i_* is the estimated binding affinity value of the *i*-th drug–target pair and *y_i_* is the actual value of the *i*-th sample. RMSE is the square root of MSE, and is also used as the metric of prediction error.

The Pearson correlation coefficient is a metric that measures the linear correlation between the predicted value *p* and the ground truth *y*:

(17) $$R=\;\frac{\sum_{i=1}^{N}{(y_{i}-\bar{y})(p}_{i}-\bar{p})}{\sqrt{\sum_{i=1}^{N}{\left(y_{i}-\bar{y}\right)}^{2}}\sqrt{\sum_{i=1}^{N}{\left(p_{i}-\bar{p}\right)}^{2}}},$$

where *p_i_* is the predicted binding affinity value of the *i*-th sample and *y_i_* is the actual value of *i*-th sample. $\bar{p}$ and $\bar{y}$ are the average of all predicted values and experimental values, respectively. The range of R values is between −1 and +1. If two variables are completely correlated, R takes +1 and if they are reversely correlated then it takes −1. If there is no correlation between variables then R takes a value of zero.

The SD in regression is the measure of imprecision (Chesher 2008), and is defined as follows:

(18) $$\mathrm{SD}=\sqrt{\frac{1}{N}\sum_{i=1}^{N}{（{(y}_{i}-({mp}_{i}+n))）}^{2}},$$

where *N* is the number of protein–ligand pairs, *y_i_* is the experimental affinity, *p_i_* is the estimated value of the *i-*th pair, and *m* and *n* are the slope and intercept of the function line between the ground truth values and predicted values.

## 3 Results

### 3.1 Comparison with competing methods

In this section, we display the performance achieved by DataDTA with the baselines on all datasets. Here, we ran each model five times to eliminate bias caused by randomness and examine the robustness of DataDTA, and the details are presented in Supplementary Table S2 and Fig. S1. We saved the optimal model obtained in these five times according to the performance on the validation set and used this optimal model on three test datasets to evaluate and compare it to existing models. We compared the predictive performance of DataDTA against five state-of-the-art deep learning prediction tools, DeepDTA (Öztürk *et al.* 2018), Pafnucy (Stepniewska-Dziubinska *et al.* 2018), TopologyNet (Cang and Wei 2017), DeepDTAF (Wang *et al.* 2021), and FusionDTA (Yuan *et al.* 2022).

To clarify the differences between our model and the above deep learning-based methods, we summarize the comparison methods below.

- DeepDTA (Öztürk *et al.* 2018) uses three-layers’ CNNs to learn the representations of the drug and protein, respectively. The feature vectors of each pair are then concatenated and fed into a multi-layer perceptron (MLP) for binding affinity prediction.
- Pafnucy (Stepniewska-Dziubinska *et al.* 2018) utilizes a 3D convolution neural network to produce a feature map of protein–ligand 3D structures, followed by dense layers for predicting affinity values.
- TopologyNet (Cang and Wei 2017) predict protein–ligand binding affinity based on protein–ligand complex 3D structures. It makes use of the element-specific persistent homology method and CNNs to generate representations of these structures for prediction.
- DeepDTAF (Wang *et al.* 2021) integrates protein sequence, protein-binding pockets, SMILES and secondary structural properties of proteins and pockets. These features are fed into embedding layers and convolution layers for DTA prediction.
- FusionDTA (Yuan *et al.* 2022) encodes drug molecules as SMILES strings, and proteins as word embeddings. Then, the LSTM layers are designed to construct the basic blocks of the encoder layer. The intermediate carriers of drug molecules and proteins are imported into the fusion layer to obtain an output carrier representation of binding affinity.
- MFR-DTA (Hua *et al.* 2023) uses amino acid embedding and word embedding for protein feature representation, and uses functional-connectivity fingerprints (FCFPs) and graph neural network (GNN) features for drugs. The proposed BioMLP module assists the model in extracting individual features of sequence elements and an Elem-feature fusion block to refine the extracted features. A Mix-Decoder block was designed to extract drug–target interaction information and predicts their binding regions.

It should be noted that DeepDTAF utilizes the pockets obtained from the protein–compound complex, which suggests that the pockets used in the DeepDTAF are the ones where the interaction occurs. However, in real applications, the protein–compound complex is commonly unavailable, therefore, we also provide the results of DeepDTAF with the predicted pockets for fair comparison. The predicted top-1 pocket structure files were retained, and the structures were converted into pocket sequences. We used 55 characters (longer sequences were truncated and shorter sequences were filled with zeros) for pocket sequences to cover approximately 90% of pockets in these datasets to satisfy the requirements of DeepDTAF and then extracted the required features to satisfy all demands described in DeepDTAF. Finally, DeepDTAF (https://github.com/KailiWang1/DeepDTAF) with the same network and parameters as Wang *et al.* (2021) was retrained and tested with the obtained data to predict the final results. Besides, we retrained the FusionDTA model (https://github.com/yuanweining/FusionDTA) and MFR-DTA model (https://github.com/JU-HuaY/MFR) with the data in our work, in which the parameters were consistent with the description in their respective publications. The models were evaluated on all test sets and the results are listed in Table 2.

**Table 2.** Prediction accuracies of DataDTA and other competing methods.

Test set	Predictor	RMSE	MSE	R	SD	CI	Complex structures		
							Native	Predicted	None
Core 2016 test dataset	Pafnucy	1.418	1.129	0.775	1.375	0.789	√	–	–
	TopologyNet	3.713	3.151	0.173	2.142	0.555	√	–	–
	DeepDTAF	1.355	1.073	0.789	1.337	0.799	√	–	–
	DeepDTA	1.443	1.148	0.749	1.445	0.771	–	–	√
	FusionDTA	1.504	1.200	0.724	1.501	0.766	–	–	√
	MFR-DTA	1.307	1.014	0.804	1.299	0.788	–	–	√
	DeepDTAF	1.536	1.253	0.716	1.520	0.760	–	√	–
	DataDTA	**1.274**	**1.012**	**0.814**	**1.265**	**0.806**	–	√	–
Test105 dataset	Pafnucy	1.392	1.169	0.750	1.176	0.782	√	–	–
	TopologyNet	4.143	3.841	0.444	1.530	0.646	√	–	–
	DeepDTAF	**1.247**	**0.966**	**0.766**	**1.149**	**0.801**	√	–	–
	DeepDTA	1.425	1.134	0.652	1.432	0.738	–	–	√
	FusionDTA	1.578	1.247	0.554	1.487	0.670	–	–	√
	MFR-DTA	1.632	1.300	0.195	1.412	0.565	–	–	√
	DeepDTAF	1.496	1.183	0.608	1.414	0.718	–	√	–
	DataDTA	1.405	1.127	0.676	1.316	0.746	–	√	–
Test71 dataset	Pafnucy	1.442	1.210	0.427	1.230	0.628	√	–	–
	TopologyNet	4.157	3.913	0.192	1.308	0.559	√	–	–
	DeepDTAF	1.273	0.998	0.480	1.194	0.656	√	–	–
	DeepDTA	1.517	1.144	0.417	1.527	0.641	–	–	√
	FusionDTA	1.222	0.971	0.525	1.158	0.680	–	–	√
	MFR-DTA	1.581	1.289	**0.605**	1.482	**0.716**	–	–	√
	DeepDTAF	1.820	1.427	0.478	1.183	0.655	–	√	–
	DataDTA	**1.220**	**0.949**	0.538	**1.146**	0.688	–	√	–

The optimal value in each column has been emphasized in bold.

Table 2 shows the comparison on the core 2016 test dataset, test71 and test105 datasets. DataDTA exhibited the best performance on the core 2016 test dataset with an R value of 0.814. The MFR-DTA model ranked second with an R value of 0.804, followed by DeepDTAF with an R value of 0.789, and Pafnucy with an R value of 0.775. On the test105 dataset, the predicted binding complex structure may introduce some bias that caused DataDTA to perform worse than Pafnucy and DeepDTAF (native complex structures). However, DataDTA achieved better performance than DeepDTA, FusionDTA, and DeepDTAF (predicted complex structures). Furthermore, it can be noticed that the performance of MFR-DTA on this dataset is inferior to the other models. DataDTA achieved competitive performance on the test71 dataset as well, but with slightly lower R and CI values compared to MFR-DTA. We also noted that although native complex structures were used in TopologyNet, the model performed poorly on three datasets. We infer that the model cannot learn valid information on this dataset. These results show the efficiency and robustness of DataDTA. We introduced two new representations, including pocket descriptors and molecular AG-FPs, to predict the binding affinity between drugs and targets. These representations complemented the input information by providing more information on local binding site of the protein and the structural properties of the compound. Moreover, our method used a novel model architecture that can better handle the protein–drug binding affinity prediction task. Unlike existing methods, we adopted a multiscale learning strategy that gradually fuses different types of information to better express the relationship between proteins and drugs. The analysis above suggested that DataDTA has unique advantages over existing methods.

**Figure 2.**
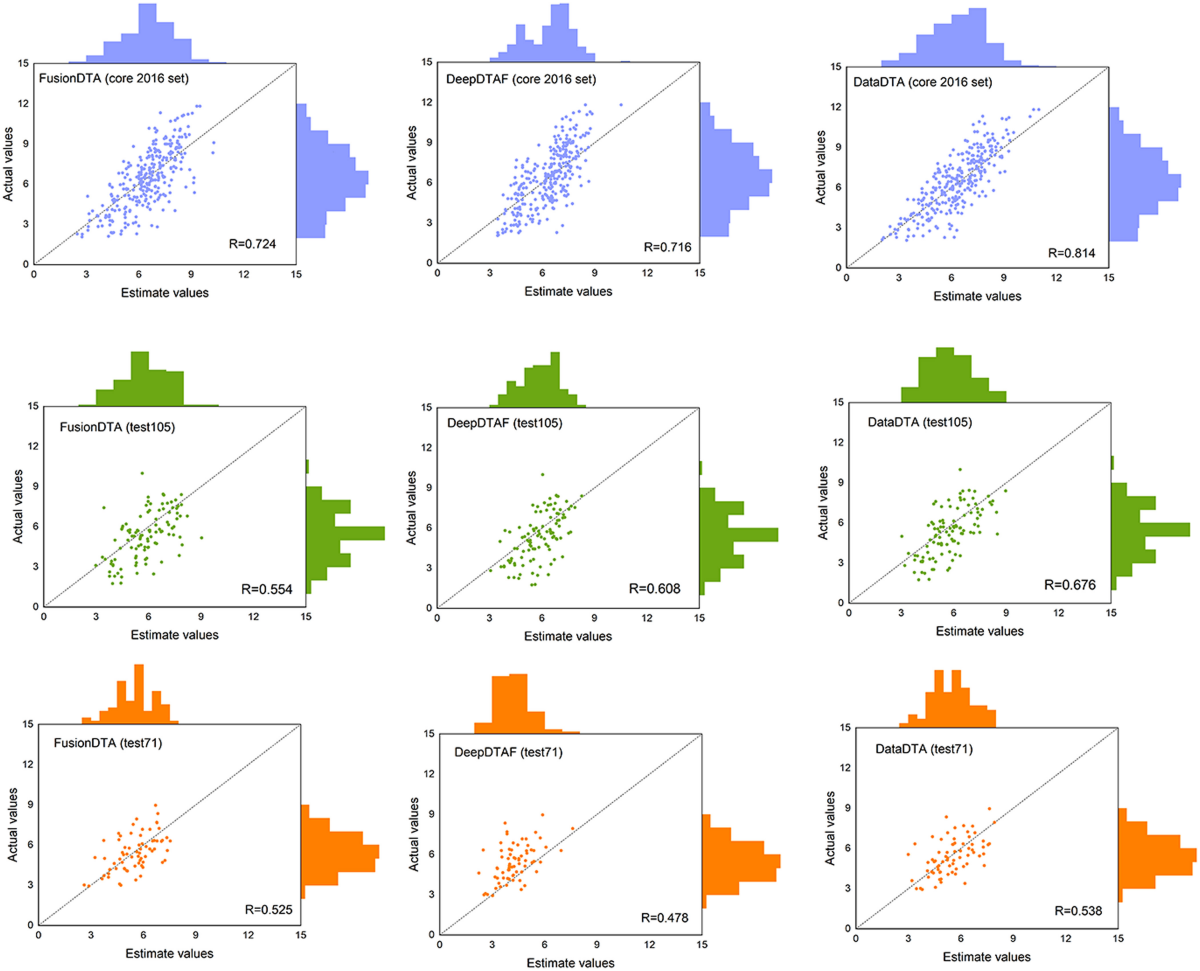
Distributions of predicted affinities on the core 2016 test dataset, test105 dataset, and test71 dataset for DeepDTAF, FusionDTA and DataDTA.

In addition, to display the comparison results more intuitively, we mapped the distributions of predicted affinities on the core 2016 test dataset, test105 dataset, and test71 dataset for DeepDTAF, FusionDTA, and DataDTA, as shown in Fig. 2.

### 3.2 The performance of various variant methods

To illustrate the effectiveness of our proposed model in various stages and the contribution of each unit to DataDTA, we conducted an ablation study by removing different parts of the model in four variants: (i) without the dual-interaction module, (ii) without the AG-FPs, (iii) without pockets, and (iv) keeping only the top-1 pocket. Similarly, we trained each ablation variant (the results for performance on the training and validation datasets are listed in Supplementary Table S3) and recorded the values for performance on the core 2016 test dataset, as presented in Table 3. We discuss the function and impact of each unit in the following sections.

**Table 3.** The performance on the core 2016 test dataset of DataDTA versus DataDTA without the dual-interaction module, without AG-FPs, without pockets, and with the top-1 pocket.

Method	RMSE	MSE	R	SD	CI
Without dual-interaction module	1.368	1.098	0.781	1.360	0.790
Without AG-FPs	1.300	1.038	0.804	1.294	0.804
Without pockets	1.314	1.042	0.799	1.308	0.801
With top-1 pocket	1.305	1.038	0.802	1.299	0.802
DataDTA	1.274	1.012	0.814	1.265	0.806

#### 3.2.1 The effects of pockets

To measure the effects of pockets on the DTA prediction, we ran the experiments on different numbers of predicted pockets. Table 3 shows that the performance of the model variants without pockets and with only the top-1 pocket was inferior to the performance of DataDTA. Specifically, the RMSE and R values of the model without pockets were 1.314 and 0.799, respectively, while those of the model with only the top-1 pocket were 1.305 and 0.802, respectively, which indicates slightly improved performance. The results show that the binding pocket descriptors we used in this work are helpful for predicting DTAs.

**Figure 3.**
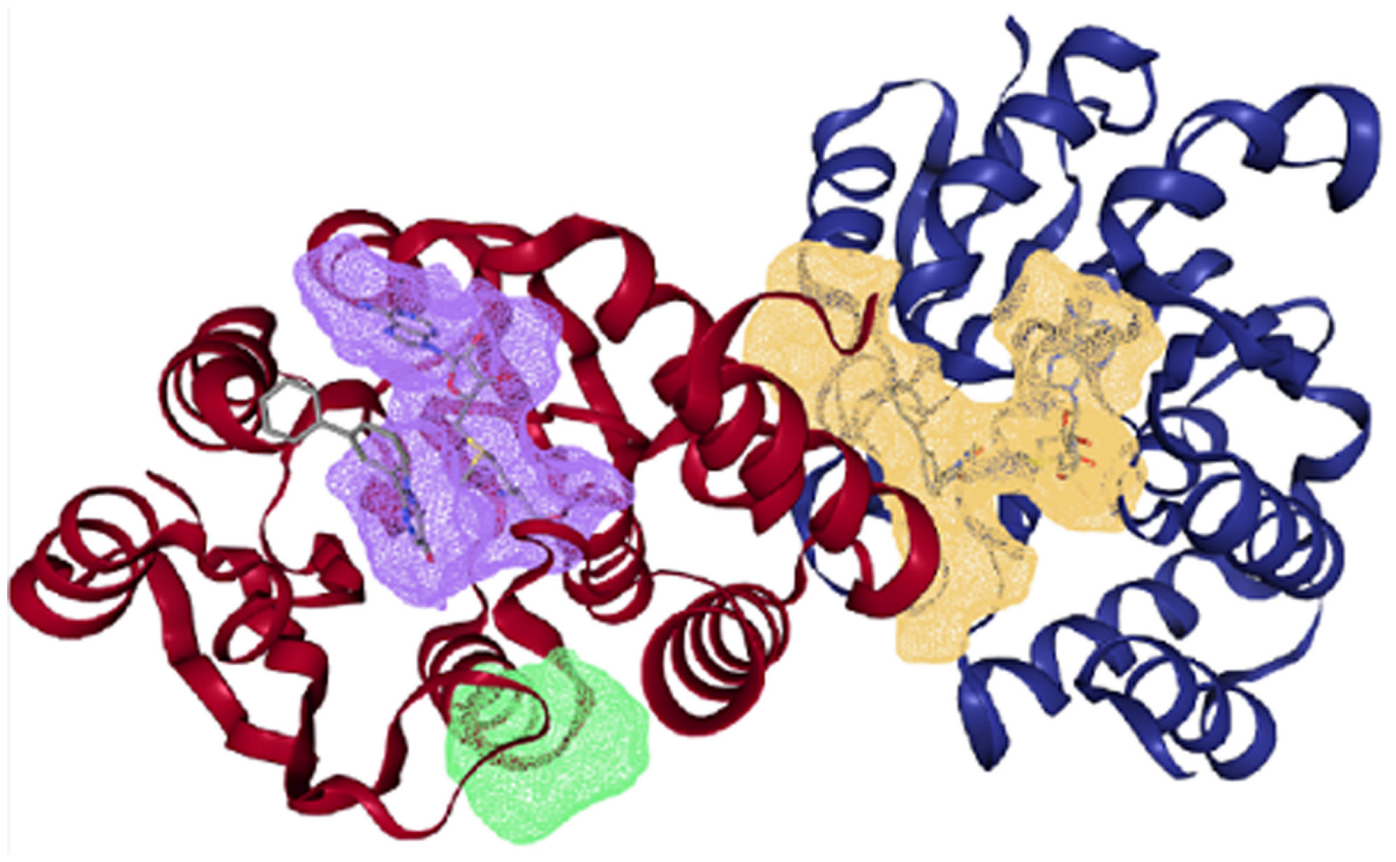
Drawing of protein, ligands, and binding pockets with PDB code: 1A0Q.

In addition, we randomly selected a binding pocket predicted by the Proteins*Plus* tool, as shown in Fig. 3. The figure shows the three-dimensional structure of the 1A0Q protein, the ligand molecules, and the three predicted pockets (orange, purple, and green areas). It can be seen that the binding regions of ligand and protein are generally located at the identified pockets, indicating that the predicted pockets are reasonable. From these results, we can say that the pocket descriptors can provide valuable information about proteins, and could be an effective protein feature representation method to predict DTAs.

#### 3.2.2 The effects of the dual-interaction module

The performance of the model without the dual-interaction module (a highway block and a multihead attention block) on the core 2016 test dataset is reported in Table 3. As shown, the CI value of the model without the dual-interaction module was 0.790, which is lower than the 0.806 CI value of DataDTA. In addition, the MSE value of the model without the dual-interaction module was 1.098, which is higher than the DataDTA MSE value of 1.012. This is also illustrated by the other values. Thus, the dual-interaction module is effective as part of the fusion stage.

We also noted that the inclusion or exclusion of the dual-interaction module has a greater impact on the model than did the features included or excluded in the other ablation experiments. Therefore, the dual-interaction module can enable exploration of potentially valuable statistics for the four features.

#### 3.2.3 The effects of AG-FPs

The algebraic graph feature AG-FPs can reduce the complexity of molecular structure while retaining basic physical and chemical information (Nguyen and Wei 2019). We noticed that most DTA prediction studies are based on the original SMILES strings for drugs, but that few are about their molecular structures (Zhao *et al.* 2019, Abbasi *et al.* 2020, Yang *et al.* 2021, Wang *et al.* 2022). Based on this observation, we studied the role of molecular structure information in this work. As seen in Table 3, both the CI and R values of the model without AG-FPs when the model was run on test dataset were 0.804, which is lower than the CI and R values of DataDTA (CI = 0.806, R = 0.814). The RMSE, MSE, and SD were 1.300, 1.038, and 1.294, respectively, which are all higher than the RMSE, MSE, and SD of DataDTA.

Taken together, it is sensible and reliable to include the AG-FPs as input drug data. It can be used as complementary information to drugs for DTA prediction.

### 3.3 Parameter analysis

In this section, we investigated the effect of varying different parameters on the performance of the proposed model. Specifically, we varied parameters including the number of characters for SMILES strings and for protein sequences, batch size, learning rate, and filter size for CNN module to evaluate their impact on model.

**Figure 4.**
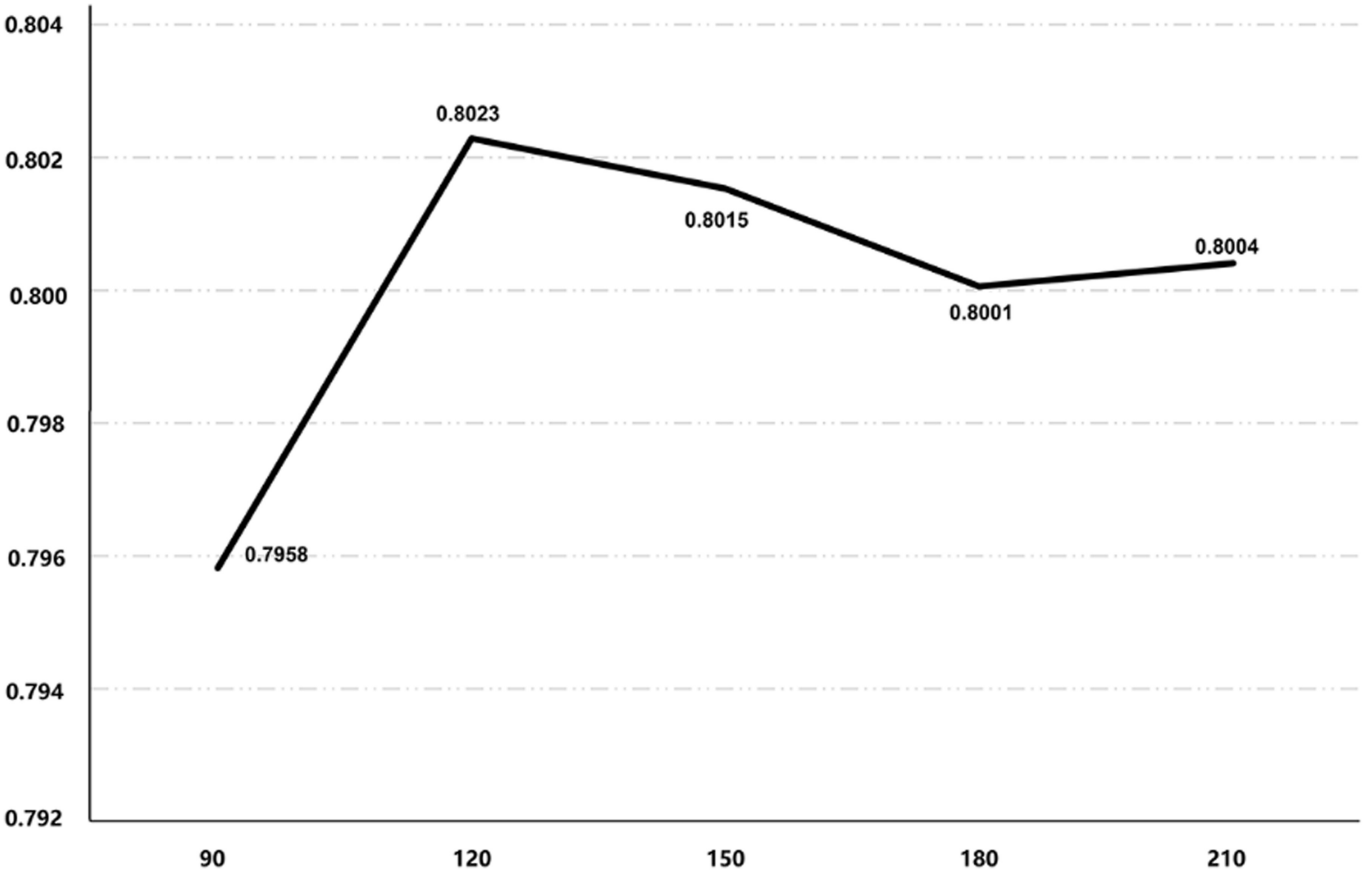
The CI values for each number of SMILES strings characters when running the model on the validation dataset.

For the number of characters for SMILES strings and for protein sequences, we observed that many studies require that the number of characters for SMILES strings and for protein sequences cover at least 90% in the datasets (Öztürk *et al.* 2018). To verify the validity of fixed input length, we compared the cut-off value of 90% length in the model with other values. According to the record of DeepDTAF (Wang *et al.* 2021), 150 characters for SMILES strings can cover around 90% of ligands and 1000 characters for sequences can cover around 90% of proteins in these datasets. Thus, we studied the effects of 90, 120, 150, 180, and 210 characters for SMILES strings in 30 steps and recorded each performance index in the Supplementary Table S4. Figure 4 shows the CI values of each length on the validation dataset. From Fig. 4 and Supplementary Table S4, it can be seen that the model’s performance gradually improves when the SMILES length is less than 120 characters. However, there is minimal variation in performance when the SMILES length reaches 120 characters or longer. In order to save computing resources, 120 characters for SMILES strings was selected in this work. Similarly, we studied the effects of 500, 1000, 1500, and 2000 amino acids for protein sequences in 500 steps and recorded each performance index in the Supplementary Table S5. We ultimately selected 1000 amino acids for protein sequences.

Additionally, we carried out experimental analysis on three commonly used parameters in deep learning: learning rate, batch size, and filter size for CNN. Several sets of experimental results were obtained by adjusting different values of these parameters and recording the performance of the model under each set of parameters. These results were listed in Supplementary Table S6. It can be seen from the table that the choice of parameters has a significant impact on the performance of the model. Specifically, a lower learning rate tends to perform better, with a learning rate of 0.0001 performing the best across all parameter settings. In contrast, the impact of batch size is minimal, with no significant difference in the performance of the model observed across values of 64, 128, and 256. Moreover, the use of larger CNN filter sizes (256) seems to improve the model’s performance. Compared with using a filter size of 128 or 512, using a filter size of 256 results in slightly higher CI and lower RMSE, MSE, and SD. Overall, selecting the right parameter combination is crucial for achieving optimal performance.

## 4 Discussion

We developed a novel predictor, DataDTA, based on multiple features and a dual-interaction module, which can effectively predict the affinities of drug–target pairs. Compared with other state-of-the-art methods, DataDTA achieved superior performance. But it can still be improved. First, DataDTA relies on binding pocket descriptors as part of input, hence it is limited by the results of the pocket prediction model. If the binding pocket can be predicted more accurately, the model may be able to further improve the prediction performance of DTA. In addition, the combined use of multihead attention and highway network affects the interpretability of the model via attention weights. Therefore, improving the flexibility and interpretability of the model is a key focus of our future research.

## 5 Conclusion

In this work, four different inputs were extracted, including protein primary sequences and drug SMILES strings, as well as binding pocket descriptors and AG-FPs. Then, the fusion strategy with a highway block and a multihead attention block was designed to integrate and capture multiscale interaction information. It is noteworthy that the predicted pocket information about key binding sites as an input is effective in predicting DTA. With the major breakthrough of protein structure prediction models and the significant development of various pocket detection tools, the attempt to use pocket descriptors in this work provides new insights for the binding affinity prediction between drugs and targets, and also provides new perspectives for other related prediction tasks in this field, such as drug–target interaction, protein–protein interaction, RNA-binding proteins prediction.

The entire datasets and source codes of DataDTA are freely available at https://github.com/YanZhu06/DataDTA. We expect that in the future, DataDTA will be used as a useful model to accelerate the process of drug discovery.

## Supplementary Material

btad560_Supplementary_Data

## Data Availability

The codes and datasets used in this article are available on Github (https://github.com/YanZhu06/DataDTA).
