# Supplementary material for: DataDTA: a multi-feature and dual-interaction aggregation framework for drug–target binding affinity prediction
Source: Bioinformatics. 2023 Sep 9;39(9):btad560. doi: 10.1093/bioinformatics/btad560 (PMC10516524; doi:10.1093/bioinformatics/btad560)
Supplement: btad560_Supplementary_Data [file btad560_supplementary_data.docx]

**DataDTA:** **a multi-feature and dual-interaction aggregation framework for drug-target binding affinity prediction**

**Supplementary Results**

**Content**

**Tables:**

**Table S1.** The finally selected optimal parameters of the model……………………………………1

**Table S2.** Predictive performance of model on five times validation tests……………………….…1

**Table S3.** The performance of DataDTA without dual-interaction module, AG-FPs, pockets and with top-1 pocket on training set and validation set…………………………………………………2

**Table S4.** Predictive performance of model for different length of SMILES strings on training set and validation set……………….………………………………………………………………2

**Table S5.** Predictive performance of model for different length of protein sequences on training set and validation set……………….………………………………………………………………3

**Table S6.** The hyperparameter tuning results on training set and validation set……………….…………………………………………………...…………………………3

**Table S1.** The finally selected optimal parameters of the model.

| Parameters | | Range |
| --- | --- | --- |
| Batch size | | 256 |
| Protein sequence length | | 1000 |
| SMILES length | | 120 |
| Epoch | | 14 |
| Early stopping | | 5 |
| Loss function | | MSE |
| Learning rate | | 1e-4 |
| CNN module | Filter size | 256 |
|  | Kernel size | 3 |
|  | Stride | 1 |
|  | Padding | 1 |
|  | Dilated size | [1, 2, 4, 8, 16] |
| Dual-interaction module | Head number | 8 |
|  | Dropout rate | 0.2 |
|  | Highway number | 1 |
| Regression prediction | Linear layers | 2 |
|  | Hidden neurons | [1024, 512, 1] |

**Table S2**. Predictive performance of model on five times validation tests.

| Dataset | Times | RMSE | MSE | R | SD | CI |
| --- | --- | --- | --- | --- | --- | --- |
| Training set | 1 | 0.665 | 0.501 | 0.937 | 0.652 | 0.895 |
|  | 2 | 0.634 | 0.632 | 0.941 | 0.632 | 0.899 |
|  | 3 | 0.582 | 0.580 | 0.951 | 0.580 | 0.908 |
|  | 4 | 0.572 | 0.570 | 0.952 | 0.570 | 0.910 |
|  | 5 | 0.634 | 0.632 | 0.941 | 0.632 | 0.899 |
|  | Average | 0.617 | 0.583 | 0.945 | 0.613 | 0.902 |
| Validation set | 1 | 1.245 | 0.965 | 0.796 | 1.242 | 0.802 |
|  | 2 | 1.273 | 0.980 | 0.792 | 1.253 | 0.801 |
|  | 3 | 1.268 | 0.984 | 0.796 | 1.244 | 0.801 |
|  | 4 | 1.258 | 0.973 | 0.797 | 1.239 | 0.801 |
|  | 5 | 1.273 | 0.980 | 0.792 | 1.253 | 0.801 |
|  | Average | 1.264 | 0.977 | 0.795 | 1.246 | 0.801 |

**Table S3.** The performance of DataDTA without dual-interaction module, AG-FPs, pockets and with top-1 pocket on training set and validation set.

| Dataset | Method | RMSE | MSE | R | SD | CI |
| --- | --- | --- | --- | --- | --- | --- |
| Training set | Without dual-interaction module | 0.868 | 0.663 | 0.891 | 0.848 | 0.858 |
|  | Without AG-FPs | 0.690 | 0.520 | 0.931 | 0.684 | 0.890 |
|  | Without pockets | 0.898 | 0.683 | 0.878 | 0.900 | 0.850 |
|  | With top-1 pocket | 0.843 | 0.639 | 0.894 | 0.837 | 0.862 |
| Validation set | Without dual-interaction module | 1.315 | 1.015 | 0.769 | 1.312 | 0.791 |
|  | Without AG-FPs | 1.326 | 1.029 | 0.770 | 1.311 | 0.790 |
|  | Without pockets | 1.339 | 1.037 | 0.765 | 1.323 | 0.788 |
|  | With top-1 pocket | 1.297 | 1.009 | 0.778 | 1.290 | 0.793 |

**Table S4.** Predictive performance of model for different length of SMILES strings on training set and validation set.

| Dataset | Number | CI | RMSE | MSE | SD | R |
| --- | --- | --- | --- | --- | --- | --- |
| Training set | 90 | 0.897 | 0.647 | 0.483 | 0.646 | 0.939 |
|  | 120 | 0.900 | 0.634 | 0.471 | 0.632 | 0.941 |
|  | 150 | 0.860 | 0.857 | 0.651 | 0.856 | 0.889 |
|  | 180 | 0.899 | 0.632 | 0.474 | 0.630 | 0.947 |
|  | 210 | 0.901 | 0.622 | 0.467 | 0.620 | 0.943 |
| Validation set | 90 | 0.796 | 1.295 | 0.995 | 1.271 | 0.786 |
|  | 120 | **0.801** | **1.273** | **0.980** | 1.254 | **0.792** |
|  | 150 | 0.786 | 1.340 | 1.045 | 1.321 | 0.766 |
|  | 180 | 0.800 | 1.277 | 0.986 | 1.256 | 0.791 |
|  | 210 | 0.800 | 1.276 | 0.986 | **1.253** | **0.792** |

**Table S5.** Predictive performance of model for different length of protein sequences on training set and validation set.

| Dataset | Number | CI | RMSE | MSE | SD | R |
| --- | --- | --- | --- | --- | --- | --- |
| Training set | 500 | 0.884 | 0.726 | 0.547 | 0.717 | 0.924 |
|  | 1000 | 0.896 | 0.652 | 0.489 | 0.650 | 0.938 |
|  | 1500 | 0.893 | 0.670 | 0.505 | 0.669 | 0.934 |
|  | 2000 | 0.889 | 0.693 | 0.521 | 0.691 | 0.929 |
| Validation set | 500 | 0.799 | 1.275 | 0.983 | 1.268 | 0.787 |
|  | 1000 | **0.803** | **1.255** | **0.973** | **1.240** | **0.797** |
|  | 1500 | 0.801 | 1.267 | 0.983 | 1.251 | 0.793 |
|  | 2000 | 0.802 | 1.263 | **0.973** | 1.250 | 0.793 |

**Table S6.** The hyperparameter tuning results on training set and validation set.

| Dataset | Learning rate | Batch size | Filter size | CI | RMSE | MSE | SD | R |
| --- | --- | --- | --- | --- | --- | --- | --- | --- |
| Training set | 0.01 | 256 | 256 | 0.724 | 1.454 | 1.137 | 1.454 | 0.629 |
|  | 0.001 | 256 | 256 | 0.910 | 0.568 | 0.416 | 0.568 | 0.953 |
|  | 0.0001 | 256 | 256 | 0.896 | 0.650 | 0.488 | 0.649 | 0.938 |
|  | 0.0001 | 64 | 256 | 0.958 | 0.296 | 0.195 | 0.295 | 0.987 |
|  | 0.0001 | 128 | 256 | 0.948 | 0.357 | 0.25 | 0.354 | 0.982 |
|  | 0.0001 | 128 | 128 | 0.916 | 0.534 | 0.397 | 0.533 | 0.959 |
|  | 0.0001 | 128 | 512 | 0.934 | 0.438 | 0.316 | 0.429 | 0.973 |
| Validation set | 0.01 | 256 | 256 | 0.727 | 1.577 | 1.246 | 1.573 | 0.643 |
|  | 0.001 | 256 | 256 | 0.798 | 1.297 | 0.992 | 1.268 | 0.787 |
|  | 0.0001 | 256 | 256 | 0.803 | 1.258 | 0.966 | 1.243 | 0.796 |
|  | 0.0001 | 64 | 256 | 0.806 | 1.243 | 0.935 | 1.226 | 0.802 |
|  | 0.0001 | 128 | 256 | **0.807** | **1.234** | **0.929** | **1.221** | **0.804** |
|  | 0.0001 | 128 | 128 | 0.797 | 1.303 | 0.991 | 1.276 | 0.784 |
|  | 0.0001 | 128 | 512 | 0.804 | 1.246 | 0.939 | 1.237 | 0.798 |
